# The Chemical Probes Portal: an expert review-based public resource to empower chemical probe assessment, selection and use

**DOI:** 10.1093/nar/gkac909

**Published:** 2022-10-21

**Authors:** Albert A Antolin, Domenico Sanfelice, Alisa Crisp, Eloy Villasclaras Fernandez, Ioan L Mica, Yi Chen, Ian Collins, Aled Edwards, Susanne Müller, Bissan Al-Lazikani, Paul Workman

**Affiliations:** Division of Cancer Therapeutics, The Institute of Cancer Research, London, SM2 5NG, UK; Department of Data Science, The Institute of Cancer Research, London, SM2 5NG, UK; Chemical Probes Portal, www.chemicalprobes.org; Division of Cancer Therapeutics, The Institute of Cancer Research, London, SM2 5NG, UK; Department of Data Science, The Institute of Cancer Research, London, SM2 5NG, UK; Chemical Probes Portal, www.chemicalprobes.org; Division of Cancer Therapeutics, The Institute of Cancer Research, London, SM2 5NG, UK; Department of Data Science, The Institute of Cancer Research, London, SM2 5NG, UK; Chemical Probes Portal, www.chemicalprobes.org; Division of Cancer Therapeutics, The Institute of Cancer Research, London, SM2 5NG, UK; Department of Data Science, The Institute of Cancer Research, London, SM2 5NG, UK; Chemical Probes Portal, www.chemicalprobes.org; Division of Cancer Therapeutics, The Institute of Cancer Research, London, SM2 5NG, UK; Department of Data Science, The Institute of Cancer Research, London, SM2 5NG, UK; Chemical Probes Portal, www.chemicalprobes.org; Division of Cancer Therapeutics, The Institute of Cancer Research, London, SM2 5NG, UK; Department of Data Science, The Institute of Cancer Research, London, SM2 5NG, UK; Chemical Probes Portal, www.chemicalprobes.org; Division of Cancer Therapeutics, The Institute of Cancer Research, London, SM2 5NG, UK; Centre for Cancer Drug Discovery, The Institute of Cancer Research, London, SM2 5NG, UK; Chemical Probes Portal, www.chemicalprobes.org; Structural Genomics Consortium, University of Toronto, Toronto, ONM5G 1L7, Canada; Chemical Probes Portal, www.chemicalprobes.org; Institute of Pharmaceutical Chemistry, Goethe University Frankfurt, Frankfurt, 60438, Germany; Structural Genomics Consortium, BMLS, Goethe University Frankfurt, Frankfurt, 60438, Germany; Chemical Probes Portal, www.chemicalprobes.org; Department of Genomics Medicine and the Institute of Data Science in Oncology, MD Anderson Cancer Center, Houston, TX, 77054, USA; Chemical Probes Portal, www.chemicalprobes.org; Division of Cancer Therapeutics, The Institute of Cancer Research, London, SM2 5NG, UK; Centre for Cancer Drug Discovery, The Institute of Cancer Research, London, SM2 5NG, UK; Chemical Probes Portal, www.chemicalprobes.org

## Abstract

We describe the Chemical Probes Portal (https://www.chemicalprobes.org/), an expert review-based public resource to empower chemical probe assessment, selection and use. Chemical probes are high-quality small-molecule reagents, often inhibitors, that are important for exploring protein function and biological mechanisms, and for validating targets for drug discovery. The publication, dissemination and use of chemical probes provide an important means to accelerate the functional annotation of proteins, the study of proteins in cell biology, physiology, and disease pathology, and to inform and enable subsequent pioneering drug discovery and development efforts. However, the widespread use of small-molecule compounds that are claimed as chemical probes but are lacking sufficient quality, especially being inadequately selective for the desired target or even broadly promiscuous in behaviour, has resulted in many erroneous conclusions in the biomedical literature. The Chemical Probes Portal was established as a public resource to aid the selection and best-practice use of chemical probes in basic and translational biomedical research. We describe the background, principles and content of the Portal and its technical development, as well as examples of its applications and use. The Chemical Probes Portal is a community resource and we therefore describe how researchers can be involved in its content and development.

## INTRODUCTION

Small-molecule chemical probes are important reagents for exploring biological mechanisms and validating targets for drug discovery, and their use represents an orthogonal and complementary approach to the application of genetic technologies (1–10). Since the sequencing of the human genome (11,12), the bottleneck has switched from gene discovery to protein annotation and there is a bias towards research on well-studied proteins and the neglect of thousands of others that remain underexplored (13,14). Experience has shown that the publication and dissemination of chemical probes, for example protein inhibitors, provide a means to accelerate protein annotation, the study of proteins in biology and disease pathology, and subsequent pioneering drug discovery and development efforts (15). However, as discussed recently (16), the widespread use of small-molecule compounds that are claimed as chemical probes but are lacking sufficient quality, especially being inadequately selective for the desired target or even broadly promiscuous in behaviour, has resulted in a large number of erroneous conclusions in the biomedical research literature – leading to wastage of precious research resources and in some cases inappropriate clinical trials. Two significant developments can be identified as helping to catalyse improvements in the development, selection and use of high-quality chemical probes. The first is the publication of consensus guidelines or ‘fitness factors’ for chemical probes (3–5) and the second is the establishment of public resources (17).

In 2015, we, as part of a consortium of international thought-leaders in chemical biology and drug discovery, published an extensive analysis of the problems around chemical tools and guidance on the appropriate use of small-molecule compounds as inhibitors or modulators of protein function to decipher biological mechanisms (18,19). We illustrated the perils of using inappropriate small-molecule reagents and how they lead to misleading results. Moreover, the typical way in which researchers find appropriate compounds for their experiments (literature and other web searches) often propagates the use of poor-quality compounds. What was required was a definitive, expert-driven resource to guide researchers on the identification and use of small-molecule probes for biological experiments. An accompanying web-based resource was created through a voluntary effort, with contributions from academia and industry, to be used as a portal for researchers to obtain expert guidance and reviews on widely used compounds. This Chemical Probes Portal (Portal) collated reviews for an initial set of compounds and has since played an important role in informing researchers regarding the usefulness of these tools. The first web resource established the concept but proved insufficient for the growth of such an important resource and only a limited number of probes and protein families were included.

Thanks to dedicated funding and global community support, we have subsequently developed a robust, extensive infrastructure that is suitable for further expansion and enhancement. Using the new infrastructure, we have created a greatly improved Portal with expanded content and capabilities (https://www.chemicalprobes.org/). Although several computational compound annotation resources exist (17), the non-profit Portal is the only resource of its kind, providing the worldwide research community with free, expert assessments of chemical probes; valuable advice on probe selection and use; and expert recommendations such as on probe concentrations, conditions and any caveats, as well as curated bioactivity data for the assessment of chemical probes. Here, we describe the new resource, together with key information about the processes for probe submission, reviewing and governance. We exemplify the expanded content and illustrate its use by the research community, and we also explain how researchers can be involved in its content and development.

## PORTAL DEVELOPMENT, INFRASRUCTURE, CONTENT, PROCESS, USE AND OUTREACH

### What is a chemical probe?

Researchers often use small-molecule inhibitors, receptor agonists, antagonists or other modulators, such as PROTACS and molecular glues, to explore biological mechanisms. If they are well-characterised and found to be potent and selective as well as proven to interact with the intended protein of interest in cells, they are termed ‘chemical probes’ that can be used with confidence by researchers to study the function of the target in cells and potentially in animal models (18,20). Compounds that do not meet these criteria can give misleading results and should not be used without experimental mitigation. Information on chemical probes can be complex, located in diverse documents and subject to biases. The Chemical Probes Portal was designed to provide the research community with expert-led advice.

### Users of the Chemical Probes Portal

The majority of those utilising the Portal are end users who can search, browse or access all expert reviews and information on the Portal website. These are usually scientists who want to consult the Portal to identify the best chemical probe(s) for their target and application of interest, check the quality of a chemical probe they might want to use, or consult other information that we provide. No registration or login is required to access any information areas of the Portal. In addition, any user may submit a suggestion for small-molecule compounds for evaluation using the Probe Submission forms.

Members of the Scientific Expert Review Panel (SERP; https://www.chemicalprobes.org/people#serp) have a dedicated portal area, protected by authentication login to preserve the scientific integrity of the data. The SERP is an international panel of academic and industry experts, who are thought leaders in chemical biology and drug discovery with experience in chemical probes. SERP members assess the quality of probes based on their own experience, consensus guidelines (3–5,8) and criteria provided (https://www.chemicalprobes.org/information-centre#probes-criteria) and they advise and comment on each probe.

### Robust processes for content and scientific review

**Figure 1.**
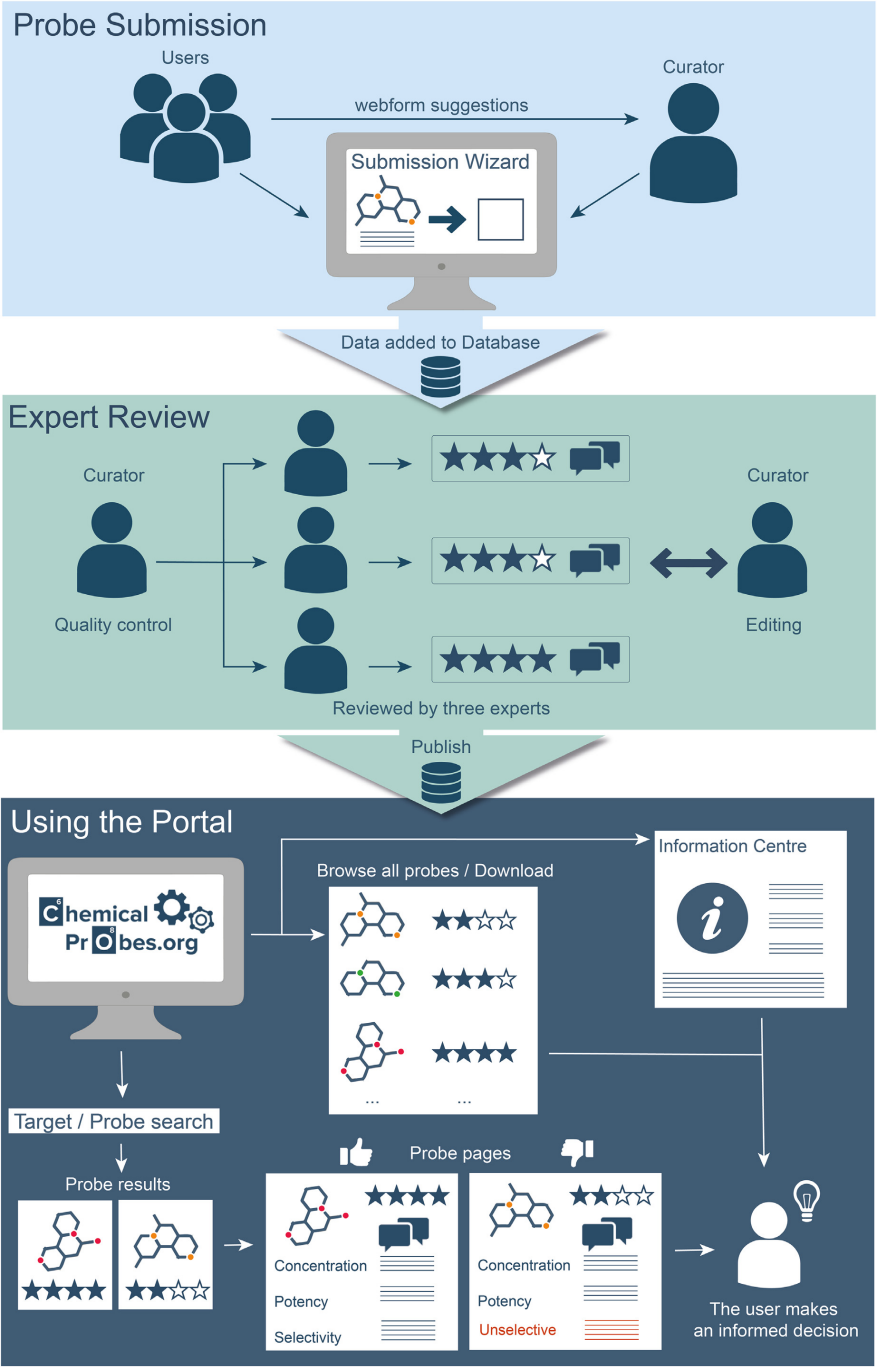
Schematic of the Chemical Probes Portal.

For a small-molecule compound to be evaluated and published on the Portal, it must first be submitted (Figure 1). Any scientist worldwide can suggest a potential chemical probe through a form on the Portal website. There are two main routes to recommend probes for the Portal. One is a minimal web form that was designed to expedite submission by requesting only very basic information on the compound. The form simply requires a chemical identifier or structure to unequivocally identify the compound, its biological target(s), and its main publication. For submitters willing to provide more information, we have implemented a user-friendly submission wizard, designed to save time by automatically completing several fields using information from the canSAR knowledgebase [http://canSAR.ai and (21)]. This wizard allows users to provide critical information, such as potency, selectivity and other important data in a systematic manner, which is essential for the reviewing process. The wizard requires that users have a login account with the Portal.

To qualify for expert review on the Portal, the proposed chemical probe must be published in the peer-reviewed scientific literature or have gone through equivalent independent scientific peer review (e.g. Donated Chemical Probes https://www.sgc-ffm.uni-frankfurt.de/#!donateview). The chemical structure must be published and the physical compound should be available.

All compounds and information proposed by users via either submission route will then be reviewed and curated by Portal curators. This will ensure that the data are complete and accurate and that the registered compound is of sufficient quality to merit expert international review.

After curation, submitted probes that pass the quality control are sent for review to members of the Scientific Expert Review Panel (SERP). For every chemical probe, the Portal curation team identifies three members from the SERP, based on their expertise, who are notified via an automated email system (Figure 1). Each SERP member provides their review through a user-friendly wizard that contains all necessary information for the assessment of the compound. SERP members are asked to provide advice on (i) the quality of the probe in terms of its potency, selectivity, and suitability for use in biological investigations; (ii) the concentrations and assay conditions in cells *in vitro*; (iii) suitability for use in animal models, commonly rodents; and (iv) any caveats or considerations that may help end users, as well as any useful additional literature references. We provide guidelines and criteria for assessing several types of chemical probes (classic modulators, PROTACs, etc.) in the Information Centre (https://www.chemicalprobes.org/information-centre#probes-criteria). The SERP reviewers each assign a star rating (one to four stars, with four stars being the highest score) for the usefulness of the probe both in cells and in animal models. Our editorial process includes selecting the appropriate SERP members based on their extensive experience in the specific target and related biology. It is their expertise that provides the greatest value. We maintain an active dialogue with the SERP members, ensuring that probes are scored in an appropriate and consistent manner, that our scoring system is clear, and at the same time learning from the SERP experience. This way, we constantly strive to enhance the reviewing process. On the rare occasions where SERP members strongly disagree, we actively moderate and always encourage SERP reviewers to support their scoring with objective data as much as possible. An average star rating is then calculated and displayed for each probe, but the individual scores from each SERP reviewer are also shown alongside any comments.

Our Rating System is transparent and public (https://www.chemicalprobes.org/information-centre#rating-system) and we recommend for use probes that have a minimum overall rating of three stars. The commentary, advice and star rating are saved in the Portal database and provided to all researchers through the Portal website. The system allows the Portal team to add further comments should additional information become available after the initial review of a compound and also has provisions to track other amendments for a submitted compound, such as editing a reviewer's response.

Portal staff, including curators, are responsible for the quality control of the information, communication with the SERP members and other day-to-day activities, including probe submission using the Portal as administrators and content managers. Portal staff and leaders additionally create scientific articles, guidelines and content which are made available through the Information Centre (see below).

The Portal leadership and development team (https://www.chemicalprobes.org/people#our-team) are responsible for the development and growth of the resource and its content. An international Board of Governors, composed of experts in their respective fields, provides oversight on the operations and strategic development of the Portal (https://www.chemicalprobes.org/people#board-of-governors).

### Current content

**Figure 2.**
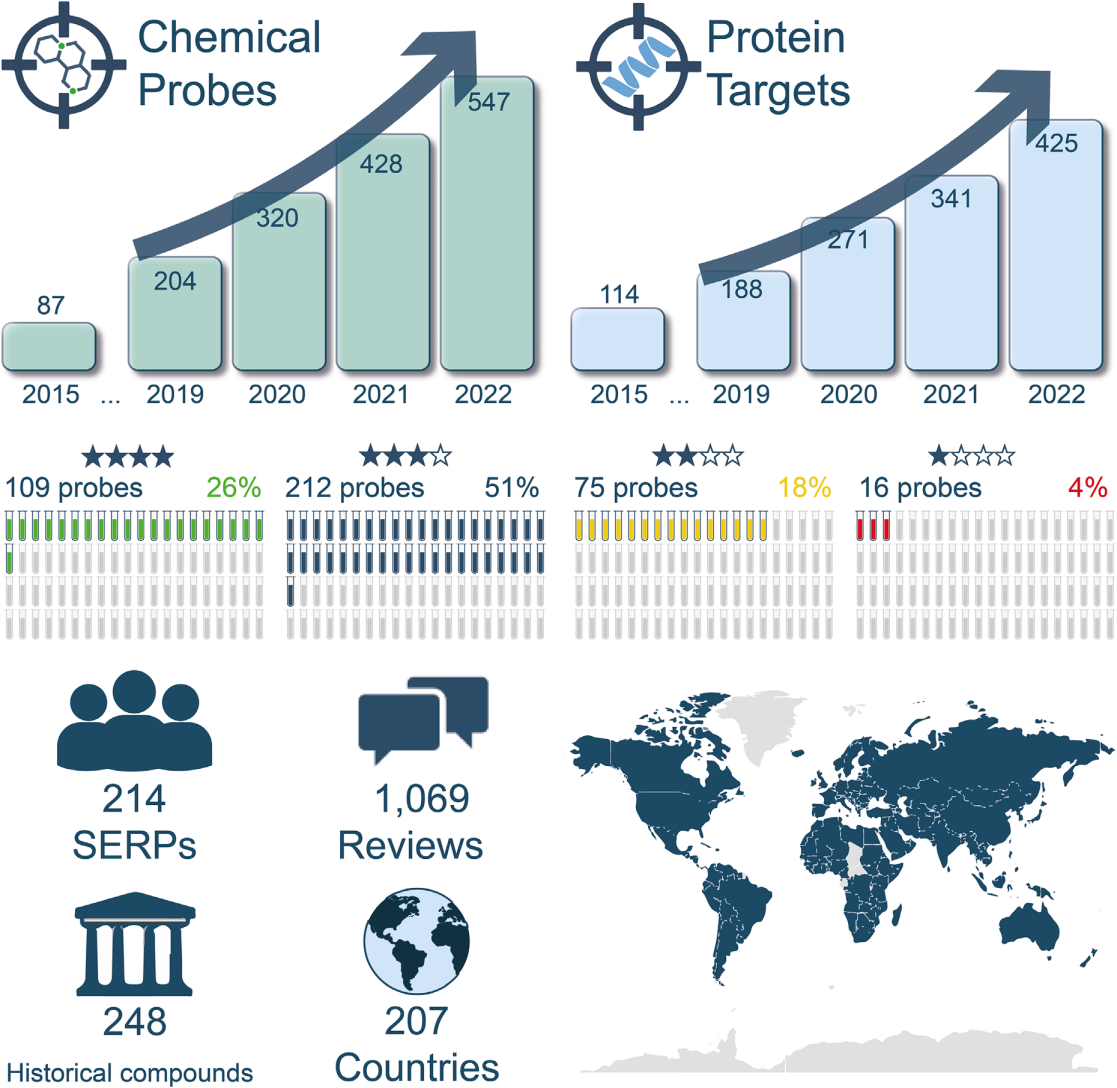
Schematic of the data growth, size and global distribution of the Portal.

The Portal now includes >500 compounds that cover over 400 protein targets and around 100 protein families (69% enzymes, Figure 2). A total of 321 chemical probes have three or more stars and are recommended by the Portal, enabling researchers to selectively study 281 particular protein targets with confidence. The compounds listed have a variety of mechanisms of action, including 390 inhibitors, 68 antagonists, 27 agonists, 34 degraders and three molecular glues.

Overall, the Portal contains 1069 expert reviews from 214 international SERP members. As mentioned earlier, in addition to the scoring, the narrative text provided by the SERP members that draws on their extensive expertise represents valuable supplementary information. SERP members frequently comment on selectivity and provide guidance on how best to use the probes in experiments and any caveats or key information that users need to know, for example, commentary on appropriate concentrations and other laboratory considerations.

In addition to the main probes described above, the Portal also lists in the Information Centre an additional 248 compounds that we refer to as ‘Historical Compounds’. These are not recommended to be used to study the function of specific proteins as they are seriously flawed or outdated and superseded. Their inclusion on the Portal is to help users avoid commonly employed but sub-optimal or misleading compounds and we have received positive feedback from users for providing this information.

The Portal is widely used by scientists from industry and academia across > 207 countries/regions.

### Searching and browsing the Portal website

**Figure 3.**
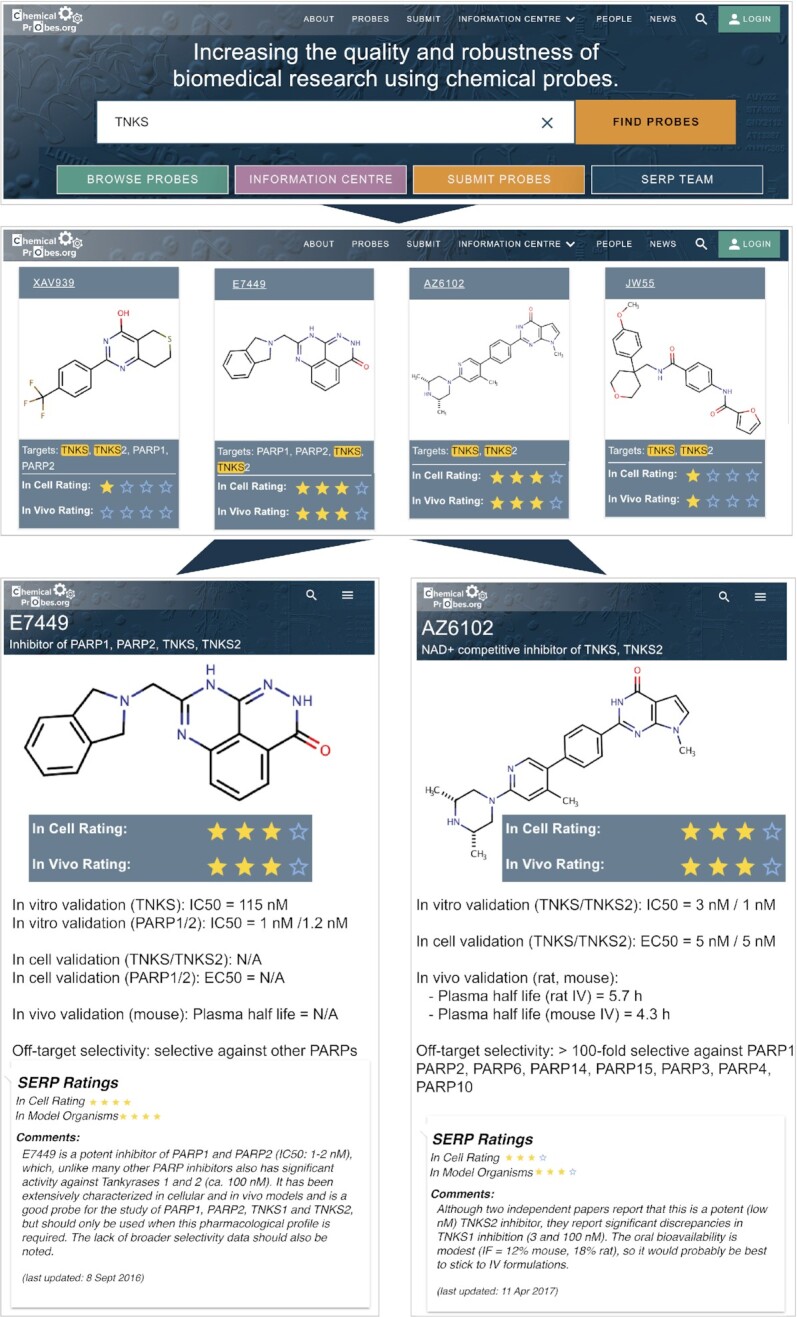
Example of using the Portal to search for chemical probes for a specific target protein.

As stated earlier, the main goal of the Portal is to help scientists identify the best chemical probe(s) to study their protein of interest. Users can search for a specific gene or protein from the landing page (Figure 1). For example, searching for tankyrase-1 (TNKS or TNKS1) yields four results (Figure 3). The search results show that probes E7449 and AZ6102 have a recommended three-star rating for use in cells whereas JW55 and XAV939 are only rated one star (Figure 3). In addition, it is obvious that while AZ6102 is a probe for TNKS1 and TNKS2, E7449 also inhibits PARP1 and PARP2 much more potently. Therefore, E7449 should only be chosen to study TNKS1/2 if simultaneous inhibition of PARP1/2 will not interfere with the phenotype under study. More details on each probe can be found by navigating to each individual probe page. For example, in the probe page for AZ6102 (Figure 3) one finds that this probe is > 100-fold selective against several PARP family members while for E7449 selectivity has been studied only in a general parylation assay and specific IC_50_ values for other PARP enzymes are not available. The expert comments from the SERP reviewers are valuable to further clarify the strengths and limitations of each probe. For example, one reviewer of E7449 mentions that ‘The lack of broader selectivity data should also be noted.’ while for AZ610 a reviewer remarks that ‘The oral bioavailability is modest, so it would probably be best to stick to IV formulations’, thus providing useful advice for *in vivo* studies. This reviewer also points out considerable potency differences in two independent assessments reported for TNKS1. Overall, the Portal helps the user make an informed choice, which in this case is likely to be that AZ6102 is the best probe among the four assessed to specifically study the tankyrases TNKS1/2.

**Figure 4.**
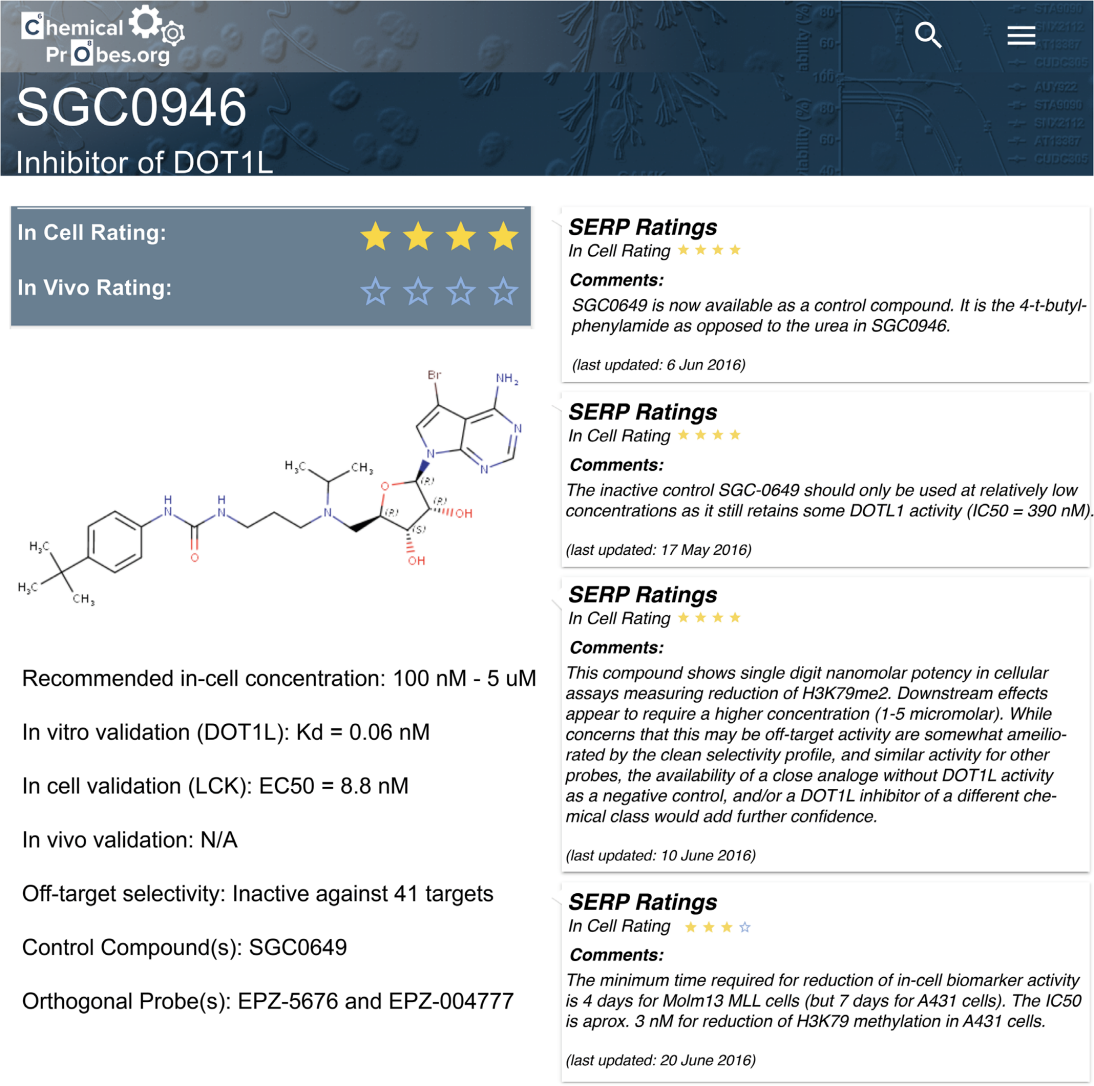
Example of a compound page.

Users can also search the Portal for a specific compound to see if it is a good choice to study a particular protein. In this case, the user would search for the compound name in the landing page search bar and, if present, select it from the results. As an example, a search for the compound SGC0946 (Figure 4) highlights that this would be an excellent chemical probe to study DOT1L in cells *in vitro* (four-star rating) but a risky choice, certainly without further information, if the user wished to study this protein *in vivo* in rodents as this probe was not assessed by reviewers for *in vivo* use. In addition, the ‘Recommended In-cell Concentration’ field is useful to avoid using excessively high compound concentrations that could compromise the selectivity of the probe (18). In this case, it is not recommended to exceed 5 μM concentration (Figure 4). Under the ‘Related Compounds’ section, the user can find available control compounds (often inactive analogues, see (22)) alongside the chemical probe of interest. These control compounds are known to be inactive, or much less potent, against the target of interest while retaining a similar chemical structure and therefore could help rule out confounding off-target effects. In this case, SGC0649 is highlighted as a control compound. Moreover, to further help rule out confounding off-target effects, the Portal also recommends the best practice of using more than one chemical probe for the same target, each with a distinct chemical structure or ‘chemotype’, known as orthogonal probe(s). In this case, two orthogonal probes are listed. EPZ-5676 has the best scores, with a four-star rating for use in cells and also a three-star rating for use *in vivo*. Therefore, EPZ-5676 would be a better alternative if the user was interested in assessing DOT1L activity in animal models, such as rodents. In this case, the SERP comments are also useful for experimental design. One expert points out that ‘The inactive control SGC-0649 should only be used at relatively low concentrations as it still retains some DOT1L activity (IC_50_ = 390 nM)’—thus helping the user select the right concentration for the control compound. Another expert highlights that for the use of SGC0946 ‘The minimum time required for reduction of in-cell biomarker activity is four days for Molm13 MLL cells (but seven days for A431 cells)’—thereby alerting the user on the different exposure times required for different cellular systems. In addition, it is worth highlighting that a link is provided to the original publication, which is always advisable to read. Links are also provided to vendors and external resources that are provided alongside molecular properties and other characteristics. Overall, there is a wealth of information in the probe pages provided through carefully curated data and expert advice which helps researchers to select chemical probes, plan their experiments and use each chemical probe in an optimal way.

### Information Centre

**Figure 5.**
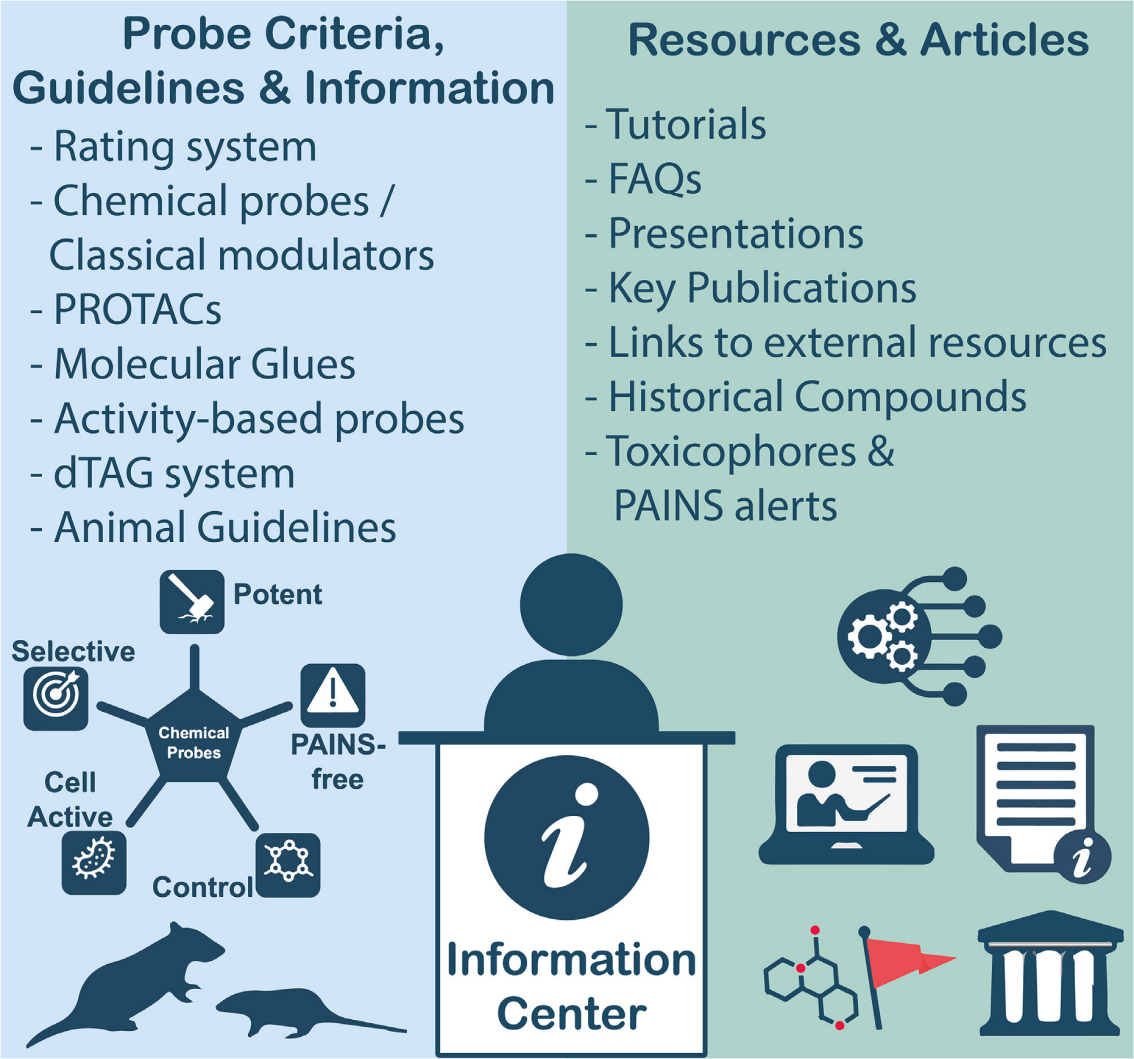
Schematic of the information available in the Information Centre.

We have devoted significant efforts to building a new Information Centre with the aim of providing the community with a centralised point to access timely, updated information about chemical probes and best practices (Figure 5). We provide probe criteria, in order to be open and transparent with the criteria that are important for chemical probe assessment, which we have organised in different sections depending on the type of chemical probe (classical modulators, PROTACs and molecular glues). We released animal model/rodent guidelines https://www.chemicalprobes.org/information-centre#animalguidelines to better support scientists that may want to develop and characterise their chemical probes for use in *in vivo* models, commonly mice. We provide additional information on topics such as PAINS and toxicophores (https://www.chemicalprobes.org/information-centre#pains) as well as links to key publications (23–25) and external resources. In the Information Centre, we respond to FAQs and explain, for example, our rating system and definition of Historical Compounds. We also have a section on activity-based probes, an emerging class of probes that we have, for now, decided not to evaluate in the Portal. Finally, we provide tutorials on how to use the Portal and links to presentations that the Portal team members have given and the slides used whenever possible. We regularly update the Information Centre, keeping on top of new probe modalities, discussions and issues in the field that are relevant to the community.

**Figure 6.**
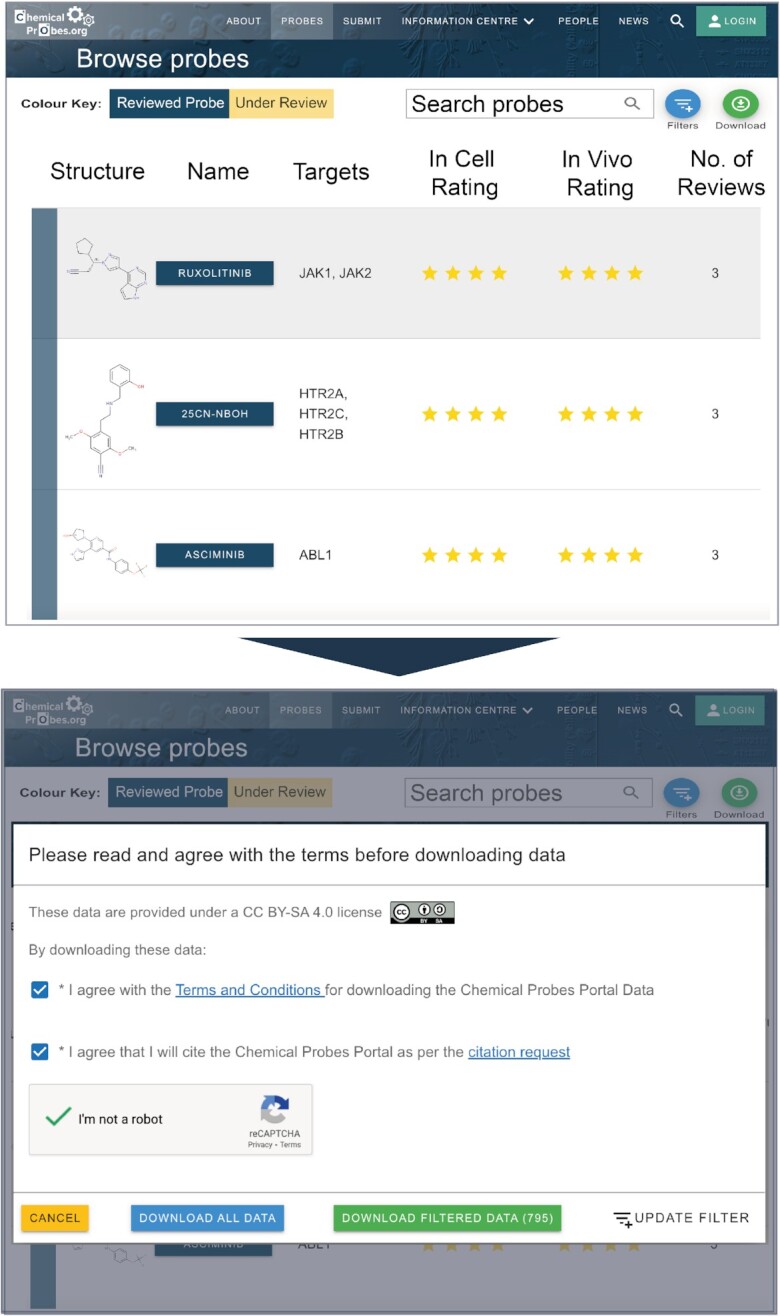
Example of the browsing and download pages of the Portal.

There is a dedicated page listing all chemical probes, which can be found under the ‘Probes’ section of the menu at the top of the website homepage (Figure 6). Users can access this page to browse the compounds listed and to filter or order the list in different ways. This section of the website enables advanced users to download all or part of the Portal data according to the filters applied. This section is particularly helpful to computational users who may want to apply our data for analysis or model training, and we have therefore designed the download to be easy to carry out and highly customizable. The data download functionality is designed considering ‘FAIR’ data principles to facilitate finding and reusing the data and is also employed by other resources that link to the Portal, such as Probes & Drugs [https://www.probes-drugs.org/home/], Probe Miner [https://probeminer.icr.ac.uk/#/] and Open Targets [https://www.opentargets.org/]. Overall, we have designed the Portal website to best serve its different uses.

### Infrastructure

The Chemical Probes Portal resource has been completely redesigned to incorporate a robust, extensible, chemically and biologically aware infrastructure that runs on an NGINX web server implemented in typescript, node.js and vue.js. The data reside in a PostgreSQL database. The data processing pipelines are written in Perl, Python and Java. To ensure its future compatibility, we implemented the underlying infrastructure to use open-source technical components where possible (e.g. PostgreSQL, Nuxt.js, Vuetify) that provide a faster, leaner product. We leverage Docker to provide a dynamic way to extend our resources only when needed, thus maintaining control of all components. The Portal uses web-development industry consensus best practices to allow for better accessibility, enhanced performance and search engine optimisation (SEO). We follow industry standards regarding colour contrasts, responsiveness of the website on various screen sizes and devices, as well as visual separation of components.

The Portal provides extensive browser compatibility for all standards-compliant browsers and adapts to work across multiple devices, including mobile devices and low-resolution screens. To help users understand the specific information and visualisations, we have implemented an extensive set of ‘in-line’ tooltips that contain glossaries and explanations. We have adopted the user-centered design approach and devoted significant efforts to optimise the website's user experience, including ensuring all wizards are user-friendly, intuitive, and easy to complete. Through interactivity with the canSAR knowledgebase (http://canSAR.ai and ref 21), we autocomplete many fields on the wizards (e.g. synonyms when a gene name is added) to reduce the time needed to complete the forms manually and thereby improve the user experience and encourage probe submission and review. Most Portal functionality and data are available without the need for log-in. However, some functionality requires credentialed use, such as the SERP review functions. Overall, we have received very positive feedback on the new Portal infrastructure from our users.

### Outreach

As well as an information resource, the Portal serves as a catalyst to change practice and improve the robustness of chemical probe use in biomedical science. To this end, we have devoted significant efforts to reach a broader audience. We regularly publish news articles and are active on social media. We provide information, references, presentations, and tutorials online and work closely with colleagues in the Target 2035 initiative (https://www.target2035.net/ and 20). We are also in active engagement with editors from major journals (16), some of whom are supporting the Portal efforts through changes to their policies on reviewing papers on chemical probes.

## FINAL REMARKS

The correct selection and use of chemical probes that are fit for purpose are important for the robustness of biomedical research. The Chemical Probes Portal provides a key resource for scientists wishing to identify the best chemical probe(s) to study their protein of interest. It is a reference used by chemical biology and drug discovery researchers but, importantly, also extends to the entire biomedical research community and indeed reaching out and providing a service for biologists who are not themselves experts in or familiar with chemical probes is a critical objective. The Portal is continuously expanding into new protein families and chemical modalities and looking for experts to support its mission to evaluate chemical probes. The Portal is cross-referenced by many multidisciplinary resources, is widely used worldwide across major academic research institutes, pharmaceutical, and biotechnology companies, and has received overwhelmingly positive feedback from our users. Moreover, our approach and criteria have been adopted by Nature journals editors to update their in-house guidelines for use in assessing submitted papers on the development and use of chemical probes, illustrating their usefulness for the community. We plan to continue working to increase the target and probe coverage of the Portal, as well as providing timely information on emerging topics and furthering our outreach programme to increase awareness and usage across all scientists employing chemical probes. We encourage the expert community to make suggestions for chemical probes and support us by dedicating some of their valuable time to evaluate chemical compounds for their usefulness as chemical probes. We also encourage all researchers, journal editors and funders to promote best practice use (16).

## DATA AVAILABILITY

The Chemical Probes Portal is freely available at https://chemicalprobes.org.
